# Global soil moisture data derived through machine learning trained with *in-situ* measurements

**DOI:** 10.1038/s41597-021-00964-1

**Published:** 2021-07-12

**Authors:** Sungmin O., Rene Orth

**Affiliations:** Max Planck Institute for Biogeochemistry, Jena, D-07745 Germany

**Keywords:** Hydrology, Hydrology

## Abstract

While soil moisture information is essential for a wide range of hydrologic and climate applications, spatially-continuous soil moisture data is only available from satellite observations or model simulations. Here we present a global, long-term dataset of soil moisture derived through machine learning trained with *in-situ* measurements, *SoMo.ml*. We train a Long Short-Term Memory (LSTM) model to extrapolate daily soil moisture dynamics in space and in time, based on *in-situ* data collected from more than 1,000 stations across the globe. *SoMo.ml* provides multi-layer soil moisture data (0–10 cm, 10–30 cm, and 30–50 cm) at 0.25° spatial and daily temporal resolution over the period 2000–2019. The performance of the resulting dataset is evaluated through cross validation and inter-comparison with existing soil moisture datasets. *SoMo.ml* performs especially well in terms of temporal dynamics, making it particularly useful for applications requiring time-varying soil moisture, such as anomaly detection and memory analyses. *SoMo.ml* complements the existing suite of modelled and satellite-based datasets given its distinct derivation, to support large-scale hydrological, meteorological, and ecological analyses.

## Abstract (summary)

**Table:** 

Measurement(s)	wetness of soil
Technology Type(s)	machine learning
Factor Type(s)	soil layer • temporal interval • geographic location
Sample Characteristic - Environment	soil

Machine-accessible metadata file describing the reported data: 10.6084/m9.figshare.14790510

## Background & Summary

Soil moisture plays a key role in land-atmosphere interactions through its control on water, energy, and carbon cycles^1,2^. Weather and climate variations are mediated by the soil moisture status^3–6^. Therefore, the spatiotemporal variations of soil moisture can influence the development and the persistence of extreme weather events such as heat waves, droughts, floods, and fires^7–11^. For these reasons, soil moisture information is required to support a wide range of research and applications, e.g. agricultural monitoring, flood and drought prediction, climate projections, and carbon cycle modelling^12^. Consequently, soil moisture is recognised as an Essential Climate Variable by the Global Climate Observing System^13^.

Despite its scientific and societal importance, large-scale long-term observations of soil moisture are scarce. There is a significant number of *in-situ* soil moisture measurement networks^14^, but they are not uniformly distributed. Satellite observations allow the derivation of global-scale soil moisture estimates; however, they represent only the top few centimetres of the soil. Moreover, satellite retrievals in areas with complex topography, dense vegetation, and frozen or snow-covered soils are challenging, leading to data gaps^15^. On the other hand, physically-based models can provide seamless soil moisture data at the global scale, but large differences exist across the models due to different and uncertain parameterisations of e.g. the spatial heterogeneity of soils and vegetation, and the non-linear relationship between soil moisture and evapotranspiration^16,17^. In summary, each source of soil moisture data has characteristic strengths and weaknesses.

Meanwhile, machine-learning (ML) presents an alternative opportunity to produce seamless soil moisture data. The usefulness of ML algorithms for soil moisture estimation or forecasting has been demonstrated in previous studies. For instance, ML is used to merge soil moisture information from different data sources^18^, to retrieve soil moisture from satellite observations like brightness temperature or backscatter^19–21^, or to simulate soil moisture using meteorological forcing^22–24^. In the last case, ML algorithms are able to ‘learn’ the complex relationship between soil moisture (target) and meteorological variables (predictors) from training data. In this way, soil moisture information can be inferred from readily observed predictor data in an empirical way without explicit knowledge of the physical behaviour of the system (e.g. land surface processes). In general, physically-based models include a range of mechanisms which are considered important and leave out others. By learning soil moisture dynamics directly from training data, ML algorithms may or may not find the same mechanisms, and hence yield different results. Consequently, the resulting soil moisture data is independent from, and can complement existing satellite-based or model-derived datasets. Similar data-driven approaches to derive gridded datasets using ML algorithms have been successfully employed in the cases of land-atmosphere fluxes^25^ and runoff ^26^.

**Fig. 1:**
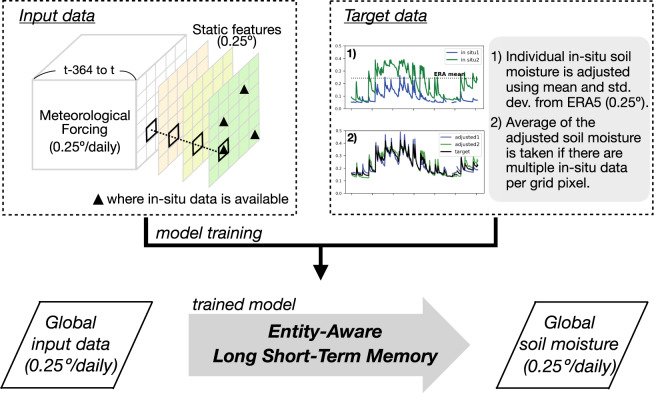
Schematic of data-driven approach to generate global-scale gridded soil moisture from *in-situ* measurements. The LSTM model is trained with meteorological data over days *t-364* to *t* and static features to simulate target soil moisture at day *t*. As *in-situ* measurements are point level data, they are adjusted using long-term mean and standard deviation of ERA5 gridded soil moisture to represent soil moisture at a 0.25 degree resolution. The model maps input-output relationships at a single grid pixel, but is trained using a combination of training data from grid pixels where *in-situ* soil moisture measurements are available.

Here we present a novel global-scale gridded soil moisture dataset generated through a data-driven approach (Fig. 1). Namely, we employ a Long Short-Term Memory neural network (LSTM)^27^ to build a soil moisture simulation model. Daily meteorological time series and static features obtained from both reanalysis and remote sensing datasets are used as predictor variables. As a target variable, we use adjusted *in-situ* soil moisture measurements from different depths obtained from the International Soil Moisture Network (ISMN)^14^ and the National Center for Monitoring and Early Warning of Natural Disasters of Brazil (CEMADEN)^28^.

*In-situ* soil moisture measurements have widely used as target variables for ML model training, often directly at a point-scale^18,20,23^. To use *in-situ* data for soil moisture modeling at a grid-scale, the limited spatial representativeness of *in-situ* data should be carefully considered. A recent study applied the extended triple collocation technique and selected only *in-situ* measurements that well represent soil moisture dynamics at the spatial scale similar to satellite footprints^21^. On the other hand, in our study, the raw point-level data are scaled to match means and variabilities of the European Centre for Medium-Range Weather Forecasts (ECMWF) ERA5 gridded soil moisture at the corresponding grid cells in order to allow seamless merging of measurements across different stations and time periods, and to estimate soil moisture at a target grid-scale. This allows training the ML model using *in-situ* data collected from a large number of stations around the globe.

Our new global soil moisture dataset, *SoMo.ml*, provides soil moisture at three different depths: 0–10 cm, 10–30 cm, and 30–50 cm, corresponding to Layer 1, Layer 2, and Layer 3, respectively. The data has a spatiotemporal resolution of 0.25° and daily, covering the period of 2000 to 2019. See Table 1 for more details.

**Table 1:** Specifications of *SoMo.ml* v1.

Data type	Gridded
Spatial Extent	Quasi-global (90° N–60° S)
Temporal coverage	2000 to 2019
Spatial Resolution	0.25° × 0.25°
Temporal Resolution	daily
Variables	Soil moisture at three layers (0–10 cm, 10–30 cm, and 30–50 cm)
Unit	Volumetric soil moisture [*m*^3^*m*^−3^]
File format	NetCDF
Key strengths	1) Global scale, long-term data. 2) Distinct data derivation compared to existing gridded soil moisture products. 3) Better agreement with *in-situ* measurements in terms of temporal soil moisture dynamics.
Limitations	1) Performance depends on *in-situ* data availability, which is low in tropical regions including Africa.
	2) Uncertainty and errors in measurements may affect the model performance.
	3) ERA5-based scaling is necessary, making long-term means and variabilities of *SoMo.ml* similar to ERA5 data.

## Methods

### Target soil moisture data preparation

Target soil moisture data at 0.25° and daily resolution for model training is constructed using the *in-situ* measurements. From the ISMN data only ‘good’ observations are selected, based on the quality flag^29^. The full list of ISMN networks involved in this study can be found in Table 2. CEMADEN provides only useful-quality data^30^. Both datasets provide sub-daily data and daily averages are computed for the days with at least six available sub-daily estimates. Stations or sensors with less than 2 months of data are discarded.

**Table 2:** List of ISMN^14^ participating networks and the number of sensors per depth considered in this study.

Network	Country	Number of sensors used (Layer1/Layer2/Layer3)
AMMA-CATCH^64^	Benin, Niger, Mali	10/6/4
ARM^65^	USA	61/42/44
BIEBRZA-S-1^66^	Poland	20/10/5
BNZ-LTER^67^	Alaska	15/13/10
CALABRIA	Italy	0/4/0
CAMPANIA^68^	Italy	0/2/0
CARBOAFRICA^69^	Sudan	1/1/0
COSMOS^70^	USA	6/36/1
CTP-SMTMN^71^	China	72/73/73
DAHRA^72^	Senegal	1/1/1
FLUXNET-AMERIFLUX^73^	USA	0/2/0
FMI^74^	Finland	24/13/8
FR-Aqui^75^	France	4/2/2
GROW^76^	UK	149/0/0
GTK	Finland	7/7/7
HiWATER-EHWSN^77^	China	166/46/46
HOBE^78^	Denmark	70/54/0
HSC-SELMACHEON	Korea	1/0/0
HYDROL-NET-PERUGIA^79^	Italy	2/0/0
ICN^80^	USA	1/1/1
IIT-KANPUR	India	1/1/1
IMA-CAN1^81^	Italy	12/0/0
IPE	Spain	2/1/0
iRON^82^	USA	11/18/0
LAB-net^83^	Chile	3/1/0
MAQU^84^	China	19/0/0
METEROBS	Italy	1/1/0
MOL-RAO^85^	Germany	2/2/1
MySMNet	Malaysia	7/0/7
OZNET^86^	Australia	35/38/38
PTSMN^87^	New Zealand	0/20/20
REMEDHUS^88^	Spain	24/0/0
RISMA^89^	Canada	58/43/44
RSMN	Romania	20/0/0
SASMAS^90^	Australia	14/14/0
SCAN^91^	USA	293/288/287
SKKU	Korea	5/5/5
SMOSMANIA^92^	France	29/30/0
SNOTEL^93^	USA	404/415/399
SOILSCAPE^94^	USA	135/145/33
SW-WHU^95^	China	3/0/0
SWEX-POLAND^96^	Poland	16/4/16
TERENO^97^	Germany	14/14/14
UDC-SMOS^98^	Germany	28/8/5
UMBRIA^68^	Italy	0/13/13
UMSUOL	Italy	1/1/1
USCRN^99^	USA	109/88/ 88
USDA-ARS^100^	USA	2/0/0
VAS	Spain	7/0/0
WEGENERNET^101^	Austria	0/12/0
WSMN^102^	UK	7/0/0

In total, we collect *in-situ* measurements from 51 ISMN networks across the globe. More detailed information can be found from https://ismn.geo.tuwien.ac.at/en/networks/.

*In-situ* measurements across the different sites are collected with various sensor types, which have different calibrations. Therefore, the means and variances of the obtained time series are not necessarily comparable, which could introduce artifacts during the LSTM training. For this reason, we adjust the mean and standard deviation of the daily *in-situ* time series to those of the respective ERA5 grid-cell soil moisture within the overlapping period. As ERA5 soil moisture is available at 0–7 cm, 7–28 cm, and 28–100 cm depths, it is vertically interpolated into the target layer depths with a depth-weighted averaging. If more than one *in-situ* measurement time series is available at the same depth within the same grid cell (0.25°), their average is taken (Fig. 1). As a result, the adjusted *in-situ* target data resembles ERA5 soil moisture in terms of mean and standard deviation, while its daily temporal variations follow the ground observations. Our approach is also based on the fact that temporal variations from point-level data have a greater areal representation compared to absolute soil moisture values^31,32^. We can therefore assume that point-level data contains sufficient information to infer soil moisture dynamics at the grid scale.

**Fig. 2:**
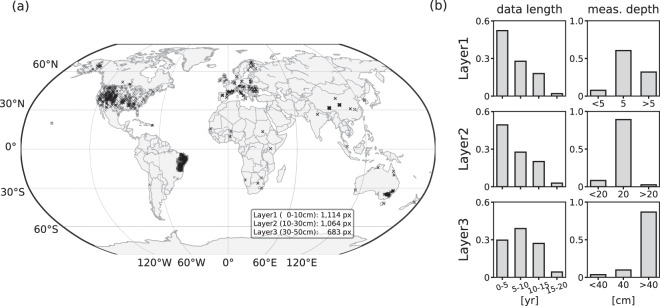
(**a**) Spatial distribution of the target soil moisture data; 1114, 1064, and 683 grid cells are available for the layers of 0–10 cm, 10–30 cm, and 30–50 cm, respectively. (**b**) Data length and measurement depths of the target soil moisture over the period of 2000–2019.

**Fig. 3:**
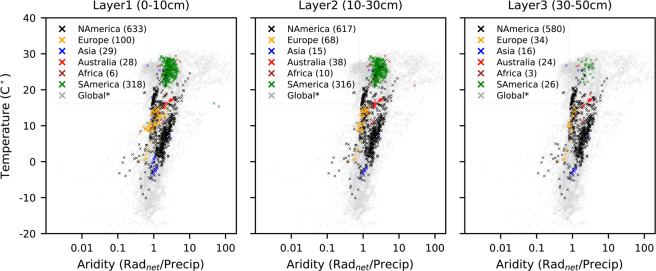
Distribution of target soil moisture across hydro-climatic regimes for each layer. The total number of target data grid cells is given for each continent. Global grid pixels are randomly sampled (5%) from all land pixels for brevity.

For each soil layer, we preferentially select the adjusted *in-situ* measurement taken at the mid-depth of the layer; i.e. 5 cm, 20 cm, and 40 cm, respectively. If no data is available at the mid-depth, the measurement taken closest to the mid-depth, and within the layer, is chosen, leading to a total of 1114, 1064, and 683 grid pixels for the three layers, respectively. The location of the grid cells with available target soil moisture is shown in Fig. 2a. Selected depths and data lengths of target soil moisture data employed for each layer are depicted in Fig. 2b. A considerable fraction of the target data is obtained from North America across diverse hydro-climatic regions (see Fig. 3). While training data from South America represents warm and semiarid regions, those from Asia mostly cover relatively cold regions.

### Model training

LSTM is a special kind of recurrent neural networks that is capable of learning long-term dependencies across time steps in sequential data^27^. It has been widely used in land surface modelling such as runoff or soil moisture simulations^23,24,33,34^. An adapted version of the LSTM architecture, *Entity-Aware LSTM*^33^, that can ingest time-varying forcing and static inputs separately is used in this study, thereby allowing the algorithm to explicitly differentiate the two different types of information.

We model soil moisture using the Entity-Aware LSTM architecture (hereafter referred to as ‘LSTM model’); the model consists of 1) 128 of hidden units, 2) one LSTM layer with one dense layer, and 3) 0.5 of dropout rate. These model hyperparameters are selected through a grid search (searching the optimal hyperparameters over the pre-defined hyperparameter space) with 5-fold cross validation. The entire dataset is split into five folds, each containing approximately 20% of the data. While the dataset is randomly split into the folds, neighbouring grid pixels are grouped into the same fold to account for spatial auto-correlation. The training of the model is performed using data from four folds, while the model validation is made with the remaining fold. This operation is repeated five times so that each fold is used once as an independent validation set, and finally the performance is averaged across the repetitions to obtain a representative estimate.

The LSTM model is trained to learn the relationship between the multiple predictor variables and the target soil moisture. The model is trained separately for each soil layer. The predictor data used for the LSTM-based soil moisture modelling is listed in Table 3. The meteorological inputs during days *t-364* to *t* are used to simulate soil moisture at day *t*; i.e. the model can establish the relationship of present soil moisture with present and past meteorological forcing over a full annual cycle. All input data are normalised using their mean and standard deviation to enhance the training efficiency^35^. We use the mean squared error divided by the standard deviation of soil moisture at each individual grid cell as a loss function. This scaling ensures comparative values of the loss function across wet and dry regions with potentially different temporal variabilities^33^.

**Table 3:** Predictor data used for the LSTM model.

	Variable	Source	Description
Dynamic	Air temperature	ERA5^36^	Daily meteorological forcing obtained from ECMWF reanalysis
	Precipitation		
	Specific humidity		
	Net surface radiation		
	Downward surface solar radiation		
	Land surface temperature		
	Soil moisture from upper layer(s) for second and third layers	SoMo.ml^50^	ML-based soil moisture produced in this study
Static	Mean precipitation	ERA5^36^	Long-term mean precipitation
	Aridity	ERA5^36^	Ratio of net radiation to precipitation
	Topography	ETOPO1^41^	Mean and standard deviation of sub-grid scale elevation values at each grid cell
	Vegetation type	GLDAS^42^	Predominant vegetation type (MODIS-derived) at each grid cell
	Soil type	GLDAS^42^	Clay, sand and silt fractions based on FAO Soil Map of the World^43^
	Soil porosity	GLDAS^42^	Soil porosity across layers, based on FAO Soil Map of the World^44^

Meteorological forcing variables are prepared from new global atmospheric reanalysis ERA5 produced by ECMWF^36^. There are several reasons why ERA5 is chosen. First, ERA5 uses large amounts and diverse kinds of observations such as synoptic station data, satellite radiance, and ground-based radar precipitation information via the 4D-Var data assimilation. Its enhanced quality as meteorological forcing, compared to its predecessor ERA-Interim, has been demonstrated through an experiment with land surface models^37^. Second, ERA5 allows the generation of long-term global-scale soil moisture data. The direct use of observations such as satellite data introduces the problem of gaps in space and time, and different or limited time periods covered by the respective variables. In this sense, the current version of *SoMo.ml* can also serve as a baseline data to evaluate performance of updated data versions in the future, e.g., by comparing with data generated from machine learning trained with purely observational data for selected variables. Finally, ERA5 is available with only a few months latency, allowing corresponding future updates of the *SoMo.ml* dataset.

For the deeper layers, soil moisture simulated from the upper layer(s) is additionally used as input data. Although the model performance of different combinations of input variables could be exhaustively compared to find ‘best’ predictors, we select meteorological forcing variables that are commonly used in physically-based modeling; the usefulness of such variables in land surface hydrologic modeling has been proven over many decades^38,39^. In addition, we assess the relative importance of predictors for the soil moisture simulations and find that land surface temperature has the greatest effect on the model performance for the top layer, while soil moisture in the upper layers(s) is the most important variable for the deeper layers. Further details are given in the following section.

For the static data, long-term mean precipitation and aridity over the period of 2000–2019 is computed using the ERA5 data^36^. Aridity is defined as the ratio of net radiation (converted into *mm*) divided by precipitation^40^. We characterise topography through mean and standard deviation of sub-grid scale elevation, as obtained from the ETOPO1 digital elevation model^41^. In addition, we use soil type and land cover information from the Global Land Data Assimilation System (GLDAS) data archive^42^. GLDAS resampled soil porosity and fractions of sand, silt, and clay from FAO datasets^43^ into 0.25° spatial resolution. The land cover is based on MODIS-derived 20-category vegetation data that uses a modified International Geosphere–Biosphere Programme classification scheme^44^. We use GLDAS Dominant Vegetation Type Data Version 2 which assigned the predominant vegetation type to each 0.25° grid cell.

#### Importance of predictors

**Fig. 4:**
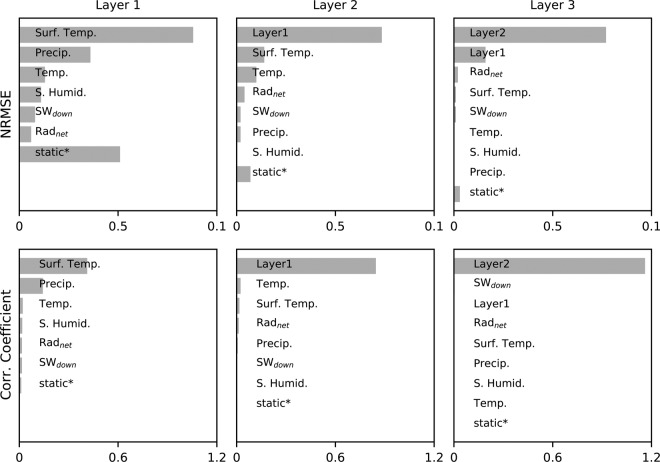
Relative importance of predictor variables for the simulated soil moisture data. We permute each predictor variable separately and compare the respective decreases in model performance; NRMSE and correlation coefficient are considered. For the static features, we permute all variables together at the same time.

The relative importance of predictor variables for the soil moisture simulation is quantified using a permutation approach. The importance is defined as the decrease in model accuracy when the time series of a particular variable is randomly permuted to remove the information contained in its temporal dynamics^45,46^. In the case of the static features, we permute all variables at the same time; each variable is randomly shuffled in space. As shown in Fig. 4, for the top layer, land surface temperature is the most significant explanatory variable among the considered meteorological forcings, followed by precipitation and 2m-temperature, in terms of both normalised root-mean-square error (NRMSE) and correlation coefficient. Land surface temperature and its diurnal amplitude has been recognised previously as a proxy for soil wetness^47–49^, confirming the LSTM results. The static data is relevant for the soil moisture performance only in terms of NRMSE. This is in line with previous findings showing that e.g. soil and vegetation types influence the spatial variability of soil moisture, but not so much the temporal dynamics^31^. While a wide range of predictor variables, including static variables, makes a significant contribution to the model performance for the first layer, (simulated) soil moisture in the upper layer(s) has the greatest effect on the model performance for the deeper layers.

### Global data generation

The LSTM model is trained using the entire training dataset which consists of the available target soil moisture data and corresponding predictor data. After establishing the internal relationships (‘learning’), the model is applied using the predictor data over a quasi-global area of 90° N–60° S at 0.25° spatial resolution. In order to account for the random initialisation of LSTM’s trainable parameters, five simulations are performed and final soil moisture values are computed as an average of the five simulations.

## Data Records

The *SoMo.ml* dataset can be accessed at figshare^50^. Three compressed files (.zip) contain data in NetCDF format for the three respective layers. An example file name is ‘SoMo.ml_v1_<LAYER>_<YYYY>.nc’, with LAYER and YYYY standing for soil moisture layer depth and year, respectively.

## Technical Validation

### Model validation

The validity of the LSTM model in soil moisture modeling is tested through 5-fold cross-validation. The simulated soil moisture for the validation is hereafter referred to as SoMo.ml*, as this simulation data differs somewhat from the actual *SoMo.ml* because it is not based on training with all available target data, but only with 80% of the data according to the 5-fold cross validation approach.

**Fig. 5:**
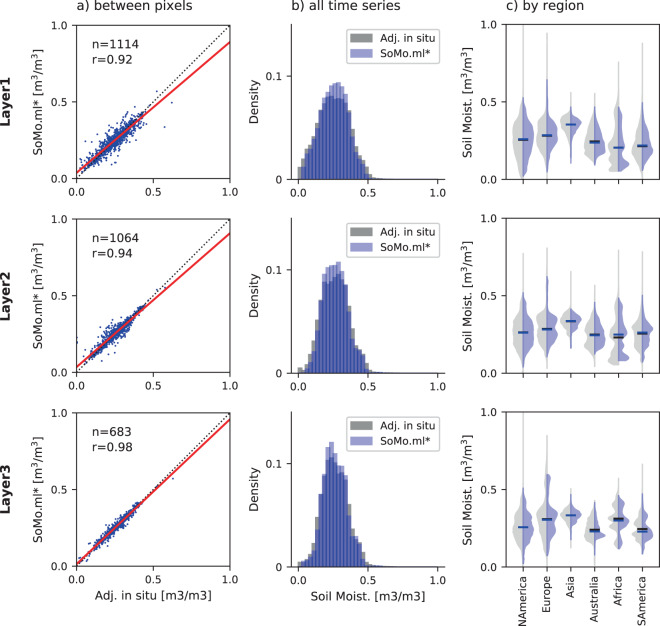
Comparison between SoMo.ml* (blue) and target soil moisture (grey) at each layer: comparison of (**a**) pixel-averaged soil moisture, (**b**) frequency distributions of daily soil moisture from all training grid cells, and (**c**) daily soil moisture from grid cells for each continent.

Figure 5a shows that the mean of SoMo.ml* at each pixel generally agrees well with that of the target data (Pearson’s r ranges between 0.92 to 0.98), indicating that the model captures spatial variations of soil moisture. The model shows somewhat better performance towards deeper layers. In Fig. 5b,frequency distributions of the entire time series of SoMo.ml* and target soil moisture are compared. Again, reasonable agreement is observed, although the simulated soil moisture exhibits smaller variability with larger minimum and smaller maximum values, as can also be seen from the slightly higher peaks of SoMo.ml*. The entire soil moisture time series are further compared for particular (sub-)continents in Fig. 5c. In terms of both distributions and medians, the model shows a satisfactory performance overall. However, relatively less agreement is observed in Africa, Australia, and South America. This is probably because the model has difficulties learning the soil moisture dynamics there as most grid cells from these regions are characterised by extreme hydro-climatic conditions (e.g. very warm or arid, see Fig. 3) for which only few *in-situ* observations are available. The (hydro-climatic) diversity of training data can significantly affect the performance of data-driven modelling; when given more diverse training data, models can acquire more complete knowledge of input-output relationships and therefore perform better across various regimes^34^. Overall, the LSTM model successfully learns soil moisture dynamics from the training data and can reproduce them at unseen locations.

### Comparison with independent *in-situ* measurements

Cross-validation (5-fold) is made through a direct grid-to-point comparison between the SoMo.ml* and the *in-situ* measurements as done in many previous studies^51–55^. This validation also enables a comparative assessment of modelled soil moisture from the LSTM with that of state-of-the-art global gridded datasets such as ERA5, GLEAM^52^, and the satellite-based ESA-CCI^15^ datasets. Established skill scores such as NRMSE, relative bias, and correlation coefficient are used to quantify the agreement with the ground truth data.

**Fig. 6:**
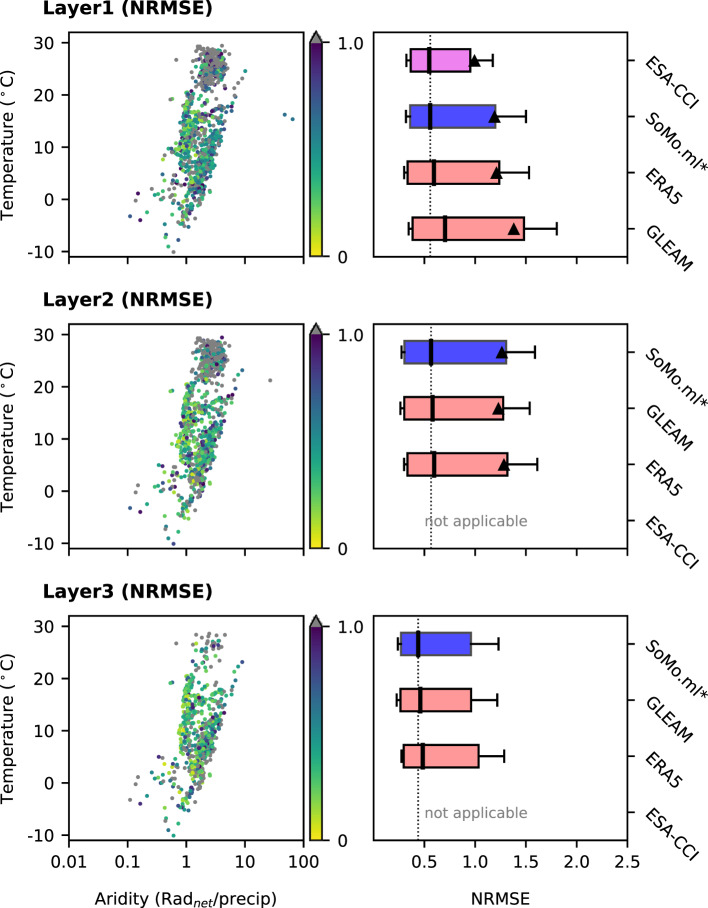
Comparison of absolute soil moisture between SoMo.ml* and *in-situ* data for each layer (top to bottom): (left) NRMSE values of SoMo.ml* at each measurement station and (right) comparison with other global gridded datasets. Triangles show mean and box plot whiskers show the 0.2 to 0.8 quantiles of the NRMSE across all measurement stations. The boxes are ranked according to the median NRMSE so that the best performing data is positioned at the top.

Figure 6 shows the distribution of the NRMSE of SoMo.ml* across climate regimes (left) and a comparison of these results with the respective performances of the reference datasets (right). NRMSE is defined as the RMSE divided by the means of ground truth. Although SoMo.ml* shows slightly higher biases at some stations over warm and arid regions, there is no clear overall climate dependency of the NRMSE. In Layer 1, while the median NRMSE of SoMo.ml* is similar to that of ESA-CCI, which shows lowest NRMSE, a wider spread of errors is observed. ERA5 and GLEAM tend to overestimate *in-situ* measurements (see Fig. S1 in Supplementary Information for relative biases), leading to slightly higher NRMSE values. In the deeper layers, where ESA-CCI is not available, NRMSE values of SoMo.ml* are slightly lower but overall similar to those of the ERA5 and GLEAM references. As a result, this comparison highlights similar deviations of absolute soil moisture values from *in-situ* measurements across the considered datasets.

**Fig. 7:**
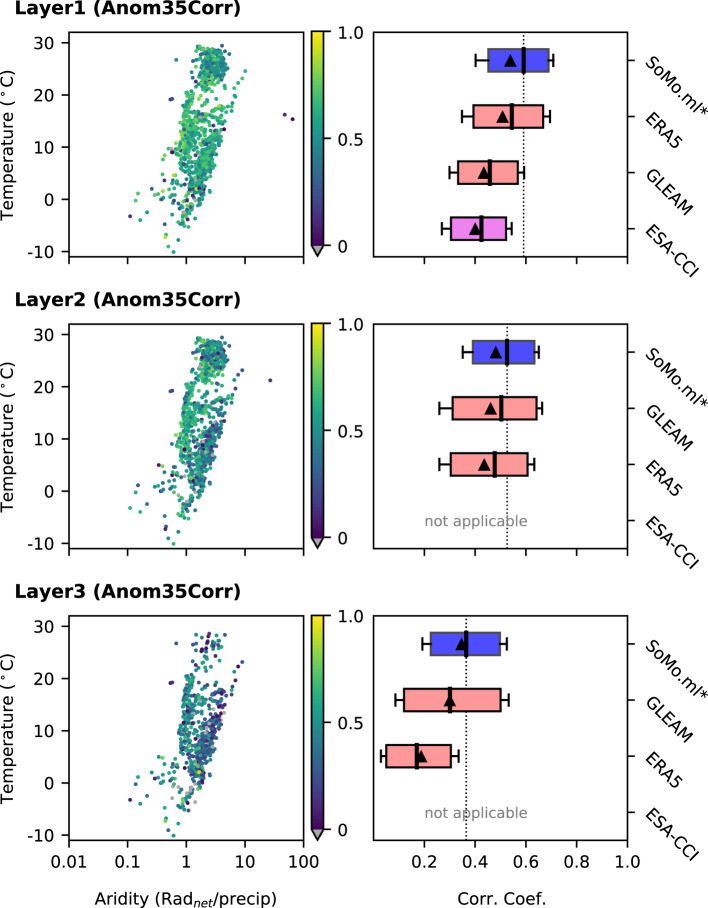
Same as in Fig. 6, but for correlation coefficient of anomalies where anomalies are determined by removing the mean of a surrounding 35-day window for each value.

Figure 7 shows results from a similar comparison, but focusing on the time-variability of the soil moisture dataset as expressed by the correlation of soil moisture anomalies with *in-situ* measurements. To exclude the impact of the seasonal cycle, we consider short-term anomalies^56,57^. For each soil moisture at day *d*, a period *P* is defined as *P* = [d-17, d + 17] (corresponding to a 5-week window). If at least 10 data are available within the period, the average soil moisture and corresponding anomaly are computed. Equations are applied to each station and a grid pixel it lies on. No pronounced climate dependency of the correlations is observed for SoMo.ml* (Fig. 7, left). Comparing with the reference datasets, SoMo.ml* outperforms them for the top layer. While overall anomaly correlations decrease in the deeper layers, also for these layers SoMo.ml* shows closer agreement with the observations than the reference datasets. The results underline the particular strength of SoMo.ml*, and likely also the actual *SoMo.ml*, to represent the temporal variability of soil moisture. This is somewhat expected; while this comparison is done against independent *in-situ* measurements, the temporal dynamics of SoMo.ml* are directly learned from (remaining) *in-situ* measurements. Similar results are obtained when using the correlations of long-term absolute soil moisture, and of anomalies derived by removing the mean daily averages (Figs. S2 and S3, respectively). We also compute the triple collocation error^58–60^, which is widely used to estimate random error variance of soil moisture data in the absence of reliable ground reference data, confirming the results from Figs. 6 and 7 and underlining the usefulness of *SoMo.ml* (Fig. S4).

Note that ESA-CCI has missing values in space and time and GLEAM is available only until 2018, such that partly different spatiotemporal data are used among datasets in the comparison. We repeat the analysis above using only data where all datasets are available and find very similar results (not shown). In summary, compared with state-of-the-art references, SoMo.ml* shows a comparable performance in terms of biases, while outperforming the other datasets in terms of temporal correlations, which highlights the benefits of using *in-situ* observation more directly in the derivation of soil moisture dataset.

### Global-scale comparison with existing gridded datasets

**Fig. 8:**
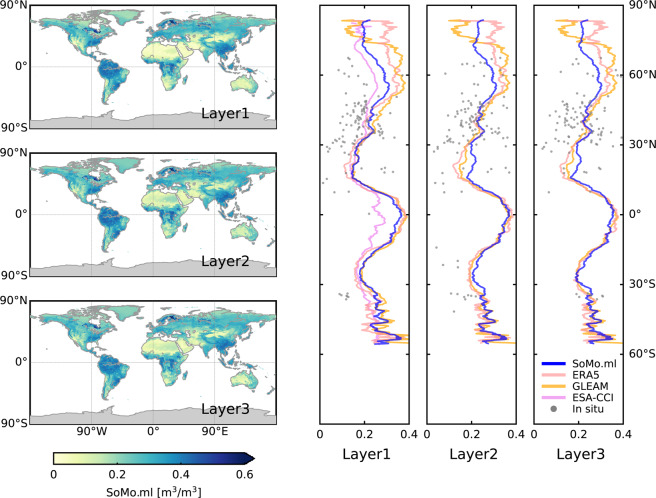
(**a**) Global maps of 20-year long-term medians of *SoMo.ml*. (**b**) Comparison of latitudinal profiles among the considered datasets. In the case of GLEAM, root-zone soil moisture is used for both Layer 2 and Layer 3.

Next, we examine the spatial patterns of *SoMo.ml* at the global scale. Figure 8a presents the median soil moisture values over the entire period. Low values in arid regions such as southwest North America, North Africa, central Asia, and Australia and high values in more humid regions such as the northern latitudes and Southeast Asia are well captured. Figure 8b compares latitudinal profile of *SoMo.ml* against that of the reference datasets (Fig. 8b). Overall, we find a satisfactory consistency between global patterns of *SoMo.ml* and the reference datasets. For instance, the highest average soil moisture occurs near the equator in the tropics, while driest soil moisture is found near 20° N. These patterns are overall well reproduced in *SoMo.ml*. This is expected to some extent because we rescale the target soil moisture using ERA5 means and standard deviations, such that the LSTM algorithm will pick up these ERA5 characteristics in locations and at time steps with available *in-situ* measurements. Nonetheless, *SoMo.ml* between 15° N and 25° N tends to be wetter than the reference datasets (over the eastern part of the Sahara desert), especially in the deeper layers. More generally, *SoMo.ml* might not properly describe soil moisture in very-arid regions, which can be related to a lack of training data from such regions (see Fig. 3). Different patterns found in ESA-CCI along the equator are mostly due to the missing data. Over very high latitudes over 60° N, we can observe relatively large differences across datasets, probably due to different freezing and thawing patterns. Meanwhile, *in-situ* measurements (not adjusted) do not show a meaningful pattern of latitudinal averages but large variability across stations and sensors, whereby it is not clear to which extent this is due to different sensor types and calibrations or due to actual moisture differences caused by heterogeneous land surface characteristics. Additional comparison among the global soil moisture datasets can be found from Figs. S5–S7 in Supplementary Information.

## Usage Notes

We present a global, multi-layer, long-term soil moisture dataset generated through a data-driven approach, and with comprehensive ground truth data. For model training, we preprocess the *in-situ* measurements to obtain more spatiotemporally consistent, grid-scale target soil moisture data by adopting mean and standard deviation from ERA5 data while preserving the observed temporal variations from the *in-situ* measurements. Any gridded soil moisture can possibly be used as a scaling reference, but the selection of reference will not affect the main characteristic of *SoMo.ml*, i.e. resembling temporal patterns of the *in-situ* measurements. Our newly generated soil moisture data outperforms other existing gridded datasets, including ERA5, in terms of daily temporal dynamics as indicated by highest temporal (anomaly) correlation with the ground observations. Nonetheless, the data quality in conditions outside the spatiotemporal range sampled within the observations is potentially uncertain. LSTM performance can be significantly affected by the (lack of) hydro-climatic diversity in the training data, even more than by the quantity of data^34^. As shown in Fig. 3, while the *in-situ* soil moisture measurements are obtained from networks worldwide, the data does not cover all globally occurring hydro-climatic conditions. Therefore, relatively high uncertainty outside the training conditions such as at high latitudes and in arid regions is expected. However, this lack of observations in particular conditions also presents a challenge to other datasets/models^57,61^. Therefore, for instance, using *SoMo.ml* within an ensemble of differently derived datasets could be a promising solution to obtain more reliable soil moisture information in these data-sparse regions^62,63^. As a result, our new soil moisture dataset is a valuable addition to the existing suite of soil moisture datasets, and can enhance future large-scale hydrologic and ecologic analyses, and also benchmark studies to evaluate land surface models and remote sensing data.

## Supplementary information

Supplementary Information

## Data Availability

The LSTM model implemented in this study and figure scripts are available from https://github.com/osungmin/SciData2021_SoMo_v1. Note that the LSTM model is built by adopting python modules obtained from https://github.com/kratzert/ealstm_regional_modeling.
