# Supplementary material for: Global soil moisture data derived through machine learning trained with in-situ measurements
Source: Sci Data. 2021 Jul 12;8:170. doi: 10.1038/s41597-021-00964-1 (PMC8275613; doi:10.1038/s41597-021-00964-1)
Supplement: Supplementary file 1 — Supplementary Information [file 41597_2021_964_MOESM1_ESM.pdf]

Global soil moisture data derived through machine learning trained with in-situ measurements

Sungmin O[1,*] **and Rene Orth**[1]

[1]Max Planck Institute for Biogeochemistry, Jena, D-07745, Germany
[*]corresponding author(s): Sungmin O (sungmino@bgc-jena.mpg.de)

Contents

Supplementary Figure 1 2

Supplementary Figure 2 3

Supplementary Figure 3 4

Supplementary Figure 4 5

Supplementary Figure 5 6

Supplementary Figure 6 7

Supplementary Figure 7 8

Reference 9

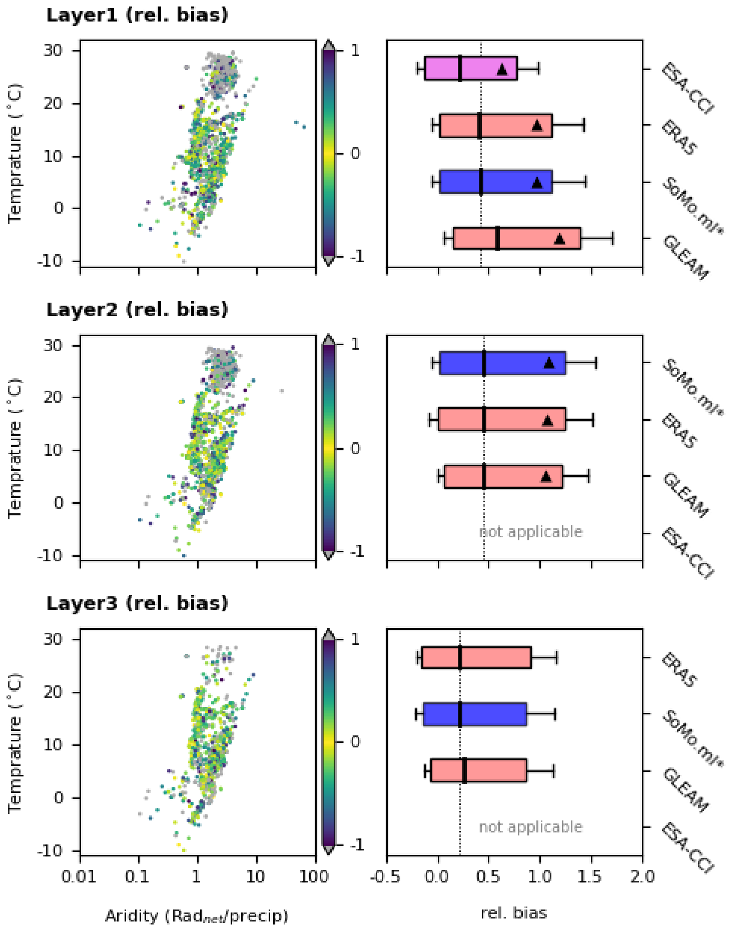

Figure S1. Same as in Fig. 6 in the main text, but for relative bias.

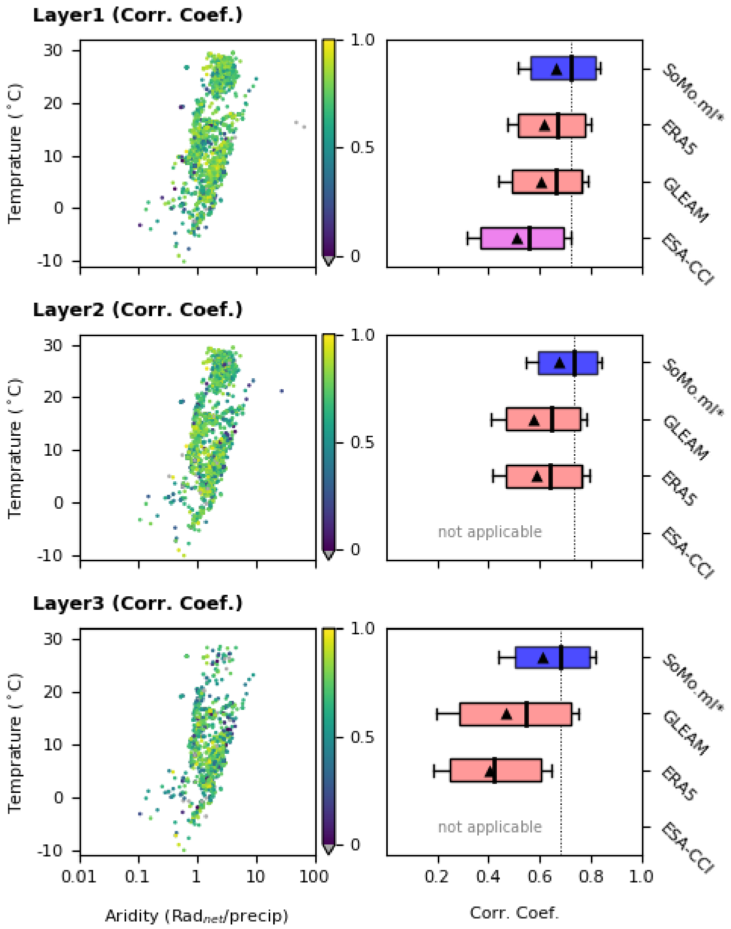

Figure S2. Same as in Fig. 6 in the main text, but for correlation coefficient of absolute soil moisture.

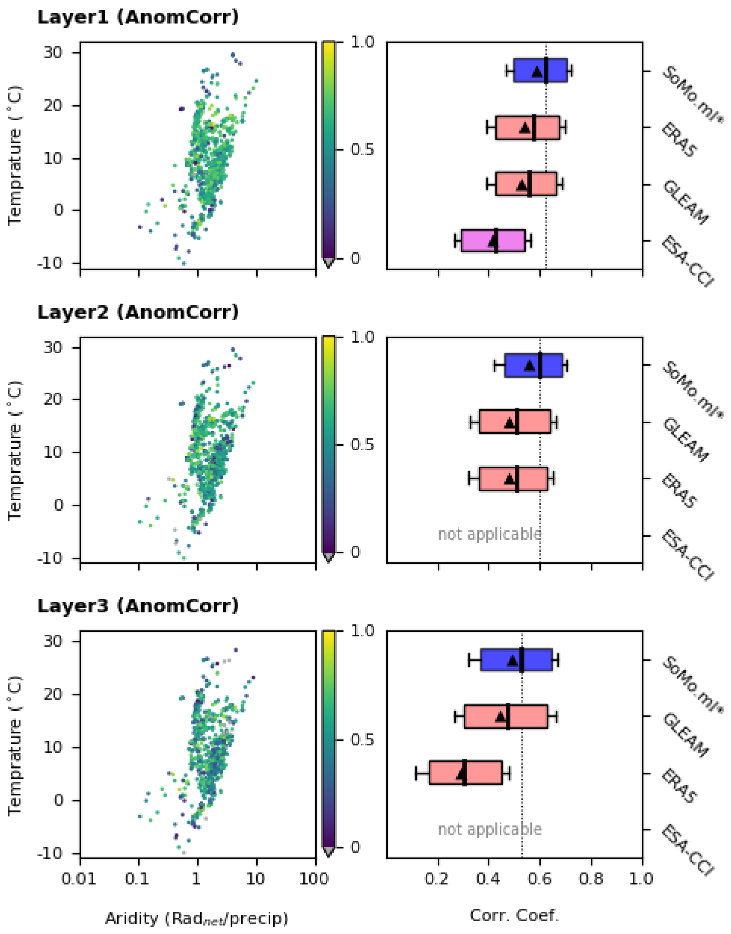

Figure S3. Same as in Fig. 6 in the main text, but for correlation coefficient of ‘climatological’ anomalies. Anomaly refers to the deviation of a specific day’s soil moisture from long-term average for the same day of the year. Only when five or more years are available, the day-of-year is included for the computation.

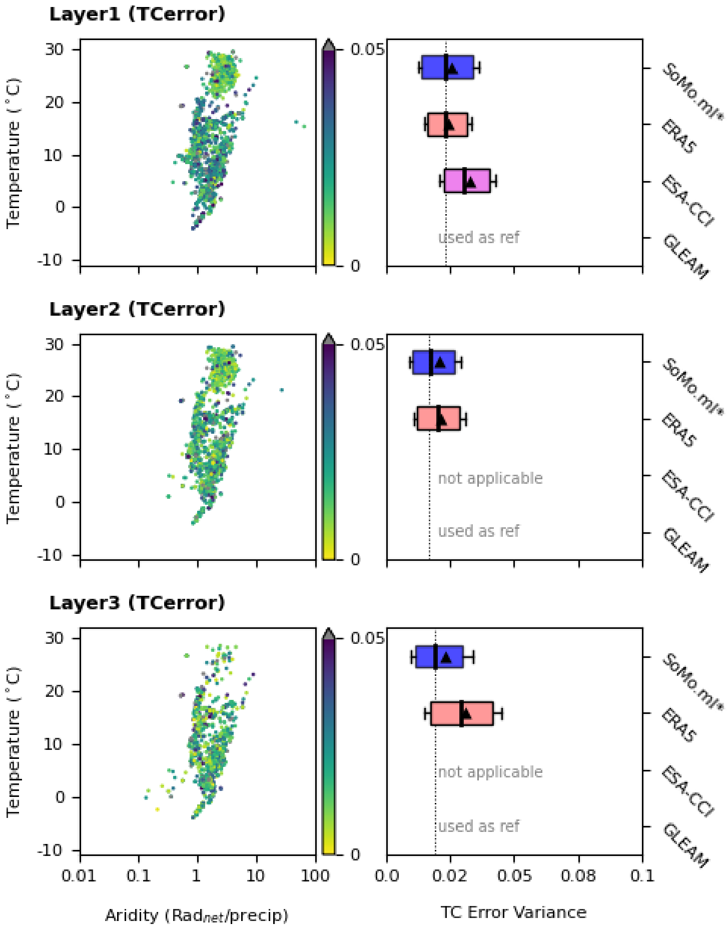

Figure S4. Same as in Fig. 6 in the main text, but for triple collocation error variance. Triple collocation analysis (TCA) is a method for estimating the random error variances of three collocated datasets with the assumption that a soil moisture estimate is related to the hypothetical unknown true soil moisture through additive and multiplicative systematic biases and random noise[1,2]. For the TAC, in-situ and GLEAM are always included and the third dataset is chosen from *SoMo.ml**, ERA5, or ESA-CCI. Python Toolbox pytesmo[3] is used for the computation.

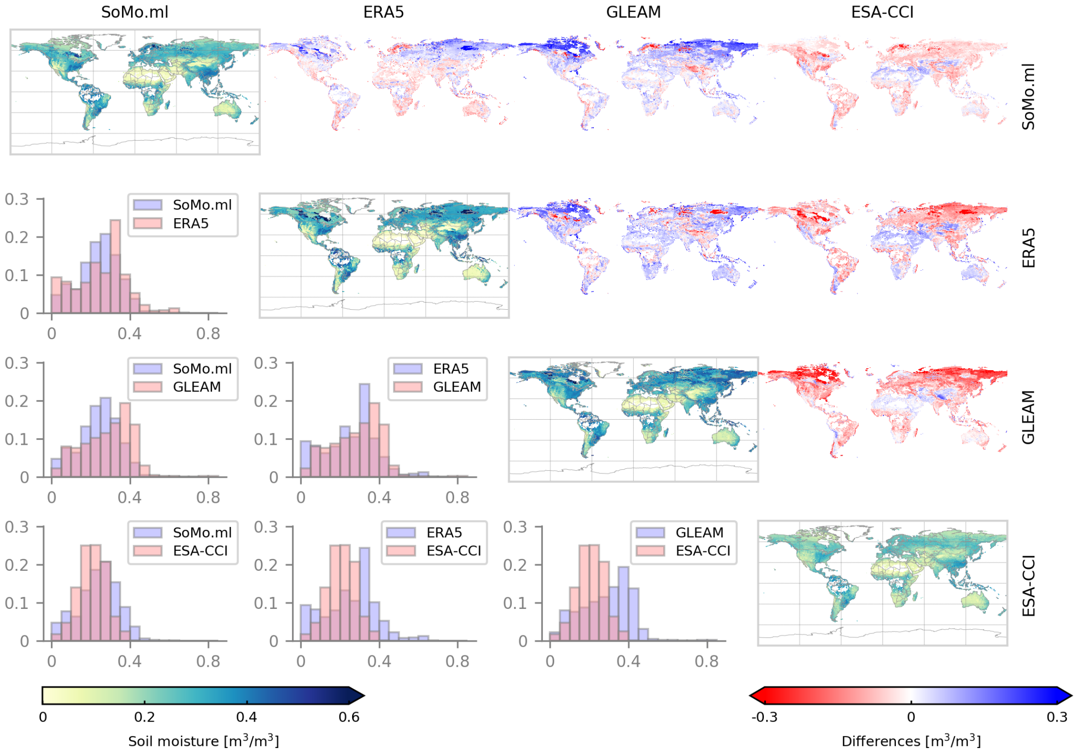

Figure S5. Comparison of the distributions of global long-term median soil moisture at Layer 1 (0-10 cm) from *SoMo.ml* and reference datasets. Along the diagonal, global maps of *SoMo.ml*, ERA5, GLEAM, and ESA-CCI are shown. Above the diagonal, maps of differences between the datasets (column minus row) are shown and below the diagonal, frequency distributions between the datasets are compared. Only grid cells where all four datasets are available are chosen for the comparison. This figure framework is adapted from Jung et al. (2019)[4].

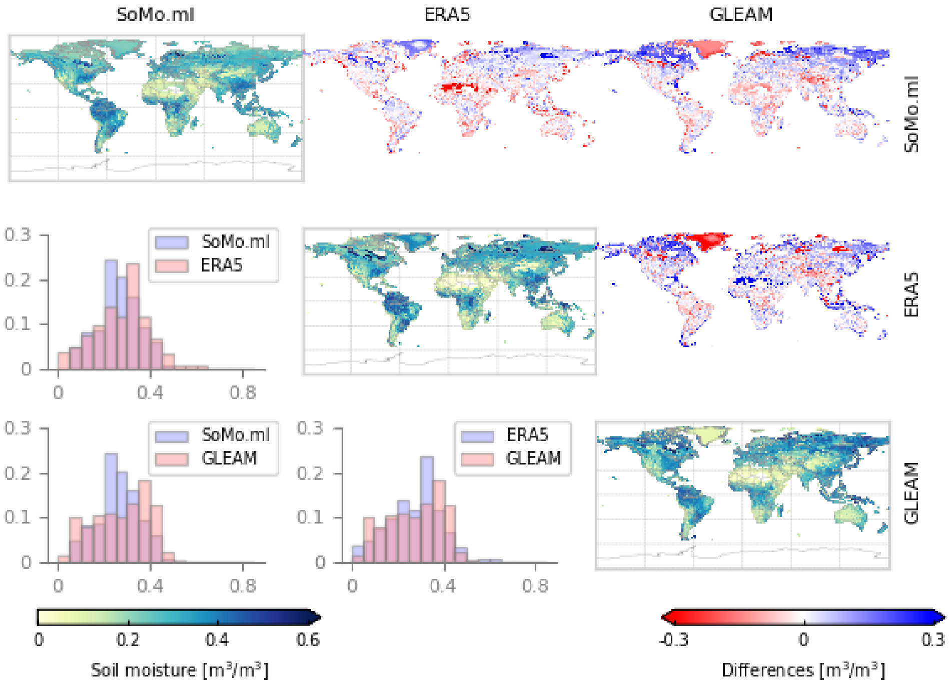

Figure S6. Same as in Fig. S5, but for Layer 2 (10-30 cm). Only grid cells where all three datasets are available are chosen for the comparison. For GLEAM, root-zone soil moisture is used.

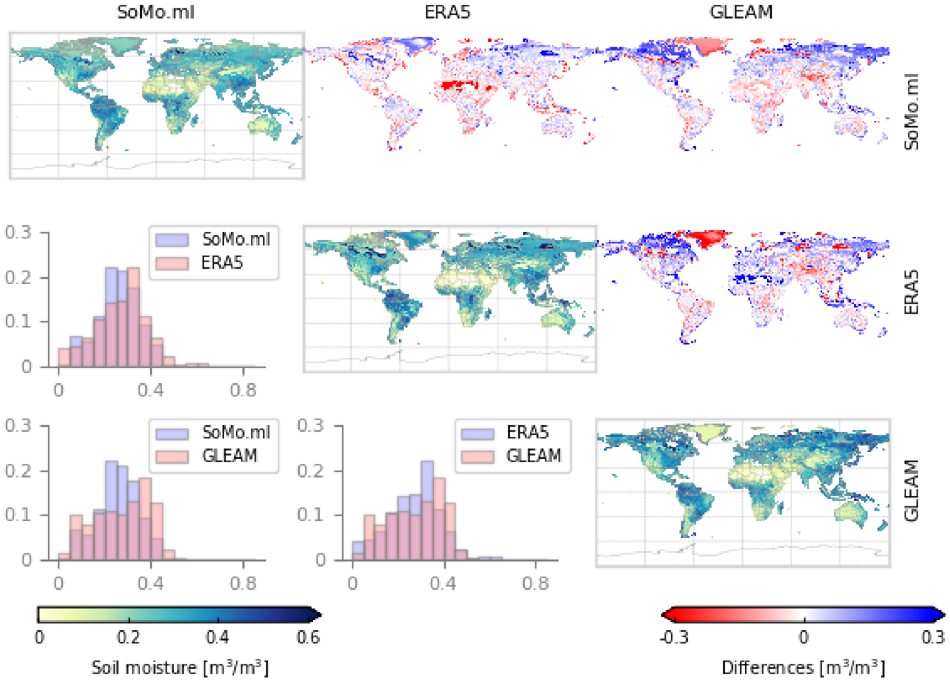

Figure S7. Same as in Fig. S5, but for Layer 3 (30-50 cm). Only grid cells where all three datasets are available are chosen for the comparison. For GLEAM, root-zone soil moisture is used.

References

1. Stoffelen, A. Toward the true near-surface wind speed: Error modeling and calibration using triple collocation. *J. Geophys. Res. Ocean.* **103**, 7755–7766, 10.1029/97JC03180 (1998).
2. Gruber, A. *et al.* Recent advances in (soil moisture) triple collocation analysis. *Int. J. Appl. Earth Obs. Geoinformation* **45**, 200–211, 10.1016/j.jag.2015.09.002 (2016).
3. Python toolbox for the evaluation of soil moisture observations v0.9.1. *Dep. Geod. Geoinformation, TU Wien* 10.5281/zenodo.4028329.
4. Jung, M. *et al.* The FLUXCOM ensemble of global land-atmosphere energy fluxes. *Sci. Data* **6**, 74, https://doi.org/10.1038/s41597-019-0076-8 (2019).
